# A predatory bivalved euarthropod from the Cambrian (Stage 3) Xiaoshiba Lagerstätte, South China

**DOI:** 10.1038/srep27709

**Published:** 2016-06-10

**Authors:** Jie Yang, Javier Ortega-Hernández, Tian Lan, Jin-bo Hou, Xi-guang Zhang

**Affiliations:** 1 Key Laboratory for Palaeobiology, Yunnan University, Kunming 650091, China; 2 Department of Zoology, University of Cambridge, Downing Street, Cambridge, CB2 3EJ, UK; 3 College of Resources and Environmental Engineering, Guizhou University, Guiyang 550003, China

## Abstract

Bivalved euarthropods represent a conspicuous component of exceptionally-preserved fossil biotas throughout the Lower Palaeozoic. However, most of these taxa are known from isolated valves, and thus there is a limited understanding of their morphological organization and palaeoecology in the context of early animal-dominated communities. The bivalved euarthropod *Clypecaris serrata* sp. nov., recovered from the Cambrian (Stage 3) Hongjingshao Formation in Kunming, southern China, is characterized by having a robust first pair of raptorial appendages that bear well-developed ventral-facing spines, paired dorsal spines on the trunk, and posteriorly oriented serrations on the anteroventral margins of both valves. The raptorial limbs of *C. serrata* were adapted for grasping prey employing a descending stroke for transporting it close the mouth, whereas the backwards-facing marginal serrations of the bivalved carapace may have helped to secure the food items during feeding. The new taxon offers novel insights on the morphology of the enigmatic genus *Clypecaris*, and indicates that the possession of paired dorsal spines is a diagnostic trait of the Family Clypecarididae within upper stem-group Euarthropoda. *C. serrata* evinces functional adaptations for an active predatory lifestyle within the context of Cambrian bivalved euarthropods, and contributes towards the better understanding of feeding diversity in early ecosystems.

The Euarthropoda – whose extant representatives include chelicerates, myriapods, crustaceans and hexapods – are ubiquitous faunal components of Lower Palaeozoic Konservat-Lagerstätten around the world. The diversity of Lower Palaeozoic total-group Euarthropoda is expressed by two fundamental morphotypes that reflect the long and complex evolutionary history of this clade: the lower stem-group euarthropods – i.e. taxa typified by a lobopodian-like construction and a one-segmented head – and the Deuteropoda, which are characterized by complete body arthrodization and a multisegmented anterior region^1^. Members of Deuteropoda are by far the most diverse and well-known euarthropods represented in exceptionally preserved fossil communities; this clade encompasses several major groups, such as the trilobitormophs (trilobites and their non-biomineralized relatives^2^,^3^), viccissicaudates (aglaspidids, xenopods and cheloniellids^1^,^4^,^5^), fuxianhuiids^6^,^7^,^8^, megacheirans^9^,^10^, and marrellomorphs^11^,^12^,^13^,^14^. The early diversity of Deuteropoda also includes an enigmatic, and most likely paraphyletic, assemblage of euarthropods typified by the presence of a bivalved carapace that covers the anterior part of the body^15^,^16^,^17^,^18^,^19^,^20^,^21^, or may extend to encompass the whole animal (e.g. *Isoxys*^18^,^22^,^23^,^24^). Although many bivalved stem-group euarthropods are known from isolated valves, articulated material with soft tissue preservation offers unique insights into the palaeobiology and evolution of these diverse organisms. In this contribution, we describe a new bivalved stem-group euarthropod from the early Cambrian (Stage 3) Xiaoshiba Lagerstätte in Kunming^8^,^25^,^26^,^27^. The exceptional preservation of completely articulated specimens reveals detailed morphological specializations for active predation expressed in the appendages and carapace, and thus contribute towards a better understanding of the palaeoecological diversity of bivalved stem-group euarthropods during the Cambrian.

## Results

### Systematic Palaeontology

Phylum (stem-group) Euarthropoda Lankester^28^ (Scion) Deuteropoda Ortega-Hernández^1^

Class and order *incertae sedis*

Family Clypecarididae Hou^29^

Genus *Clypecaris* Hou^29^

### Constituent taxa

*Clypecaris pteroidea* Hou^29^ (type species), *C. serrata* sp. nov.

### Emended diagnosis

Small bivalved euarthropods with stalked eyes, uniramous first appendages, and sub-ovate valves that cover the anterior half of the body. Trunk with at least 20 ‘thoracic’ tergites bearing biramous appendages, and three abdominal tergites without limbs. Trunk tergites on anterior half of the body bear paired series of dorsal spines connected to the trunk by round sockets. Posterior body termination consists of subconical telson with a pair of acuminate tail flukes that do not overlap at their proximal bases. Tail flukes with elevated longitudinal ridge, and elongate setae on their inner margins.

### Remarks

The definition of *Clypecaris* has been emended from Hou^29^ to reflect the morphology of its constituent taxa more accurately based on the new fossil material from the Xiaoshiba Konservat-Lagerstätte. These changes also clarify the distinction between clypecaridids and members of the morphologically similar Family Waptiidae^30^, which differ in the number of trunk tergites and the structure of the tail flukes (see discussion below). We follow Hou *et al.*^31^ (see also ref. 32) in the systematic treatment of the taxon *Ercaicunia multinodosa*^33^ as a synonym of *Clypecaris pteroidea*^29^.

***Clypecaris serrata***sp. nov.

### Etymology

*Serra* (Latin), saw, referencing the backward pointing spines along both anteroventral margins of the bivalved carapace.

### Diagnosis

*Clypecaris* with robust first appendage pair with eight podomeres, typified by well-developed antero-ventral facing spines. Valves with up to four spine-like processes on antero-ventral margins.

### Type material

**Figure 1:**
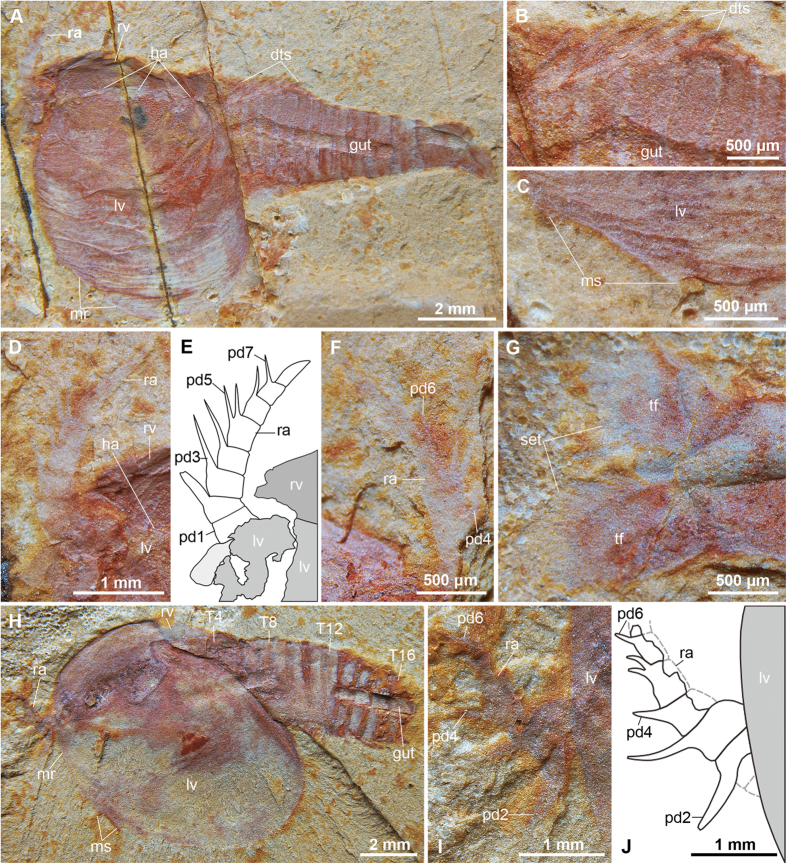
*Clypecaris serrata* sp. nov. from the early Cambrian (Stage 3) Xiaoshiba Lagerstätte in Kunming, southern China. (**A–E)**, YKLP 12325a, holotype: (**A**) left-side view of a complete individual showing bivalved carapace with compactional wrinkles; (**B**) close-up of long dorsal trunk spines, (**C**) magnification of two incomplete marginal spines; (**D**) close-up of the raptorial appendage showing six spine-bearing podomeres; (**E**) interpretative drawing of panel (**D**). (**F,G**) YKLP 12325b, holotype counterpart: (**F**) magnification of raptorial appendage; (**G**) magnification of tail flukes with elongate setae. (**H**) YKLP 12326, paratype, left-side view of an individual with at least 16 trunk tergites, showing raptorial appendages but missing the posterior end of the trunk. (**I**) detail of (**H**) showing the raptorial appendage with spine-bearing podomeres. (**J**) interpretative drawing of panel (**I**). Abbreviations: pd*n*, podomere number in raptorial appendages; dts, dorsal trunk spine; gut, digestive tract; ha, hinge articulation; lv, left valve; mr, marginal rim; ms, marginal spine; ra, raptorial appendage; rv, right valve; set, setae; tf, tail fluke; T*n*, observable trunk tergites.

**Figure 2:**
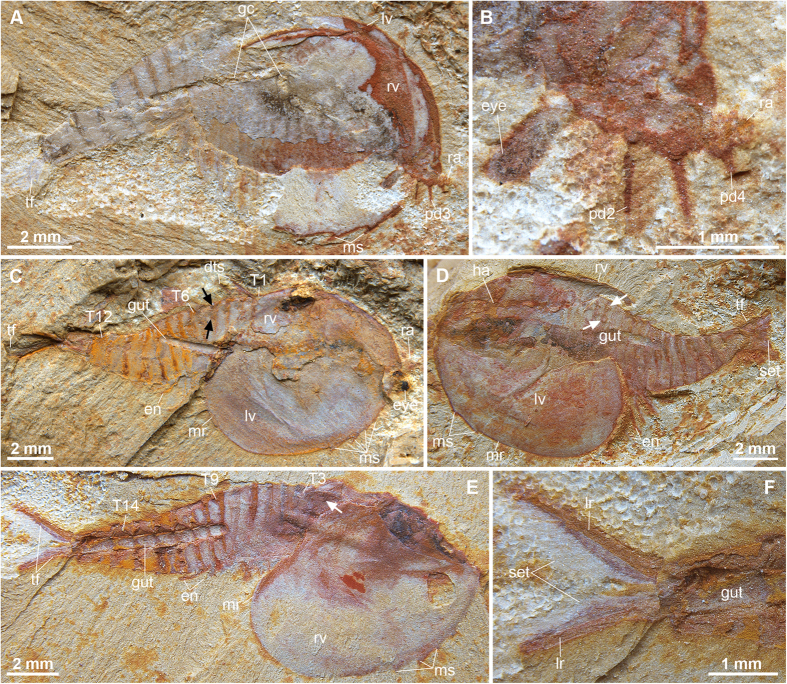
*Clypecaris serrata* sp. nov. from the early Cambrian (Stage 3) Xiaoshiba Lagerstätte. (**A,B**) YKLP 12327: (**A**) right-side view of a nearly complete individual with preserved eye. (**B**) magnification of panel (**A**) showing the right stalked eye and raptorial appendage with spine-bearing podomeres. (**C**) YKLP 12330, paratype, right-side view of a nearly complete individual showing 14 trunk tergites, a stalked eye stretching out of the carapace, inner surface of the left valve, dorsal trunk spines, spine sockets (arrowed), and four serrations on the anteroventral carapace margins. (**D**) YKLP 12331, oblique left-side view of a complete individual, showing hinge articulation, paired dorsal trunk spine sockets (arrowed) and some limbs. (**E,F**) YKLP 12332, (**E**) right-side view of a complete individual showing 15 trunk tergites. (**F**) magnification of (**E**) showing the long setae on the acuminate tail flukes. Abbreviations: en, endopod; ex, exopod; eye, stalked eye; gc, gut content; lr, longitudinal ridge; others as in Fig. 1.

Holotype YKLP 12325, a complete specimen with the first pair of raptorial appendages preserved (Fig. 1A); paratypes YKLP 12326 displaying raptorial limbs (Fig. 1H) and YKLP 12330 bearing trunk limbs (Fig. 2C). YKLP: abbreviation of the Key Laboratory for Palaeobiology, Yunnan University.

### Description

**Figure 3:**
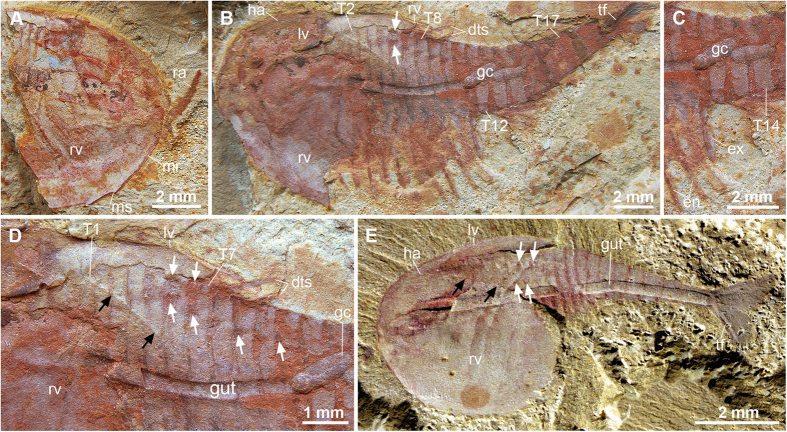
*Clypecaris serrata* sp. nov. and *C. pteroidea* from the Cambrian Stage 3 of eastern Yunnan. (**A–D**) *C. serrata* sp. nov. from the Xiaoshiba Lagerstätte. (**A**) YKLP 12329, partially preserved right valve with raptorial appendage in extended position. (**B–D**) YKLP 12328: (**B**) oblique dorsal view of a complete individual showing the hinge articulation, 18 ring-like trunk tergies with some anteriorly situated between the two valves, biramous limbs, and dorsal trunk spines (white arrowed). (**C**) magnification of panel B showing the trunk limbs and three-dimensionally preserved gut contents. (**D**) magnification of (**B**) showing trunk tergites between the two valves with visible dorsoposterior margin of left valve (black arrowed), paired dorsal spines, spine sockets (white arrowed). (**E**) *C. pteroidea* from the Chengjiang biota YKLP 13970 (courtesy Derek Siveter), showing short hinge articulation, spine sockets (white arrowed) between two valves (black arrows indicate the dorsoposterior margin of left valve). The lack of serrations of the anteroventral carapace margins of *C. pteroidea* dinstinguishes this taxon from *C. serrata*. Abbreviations: as in Figs 1and 2.

**Figure 4:**
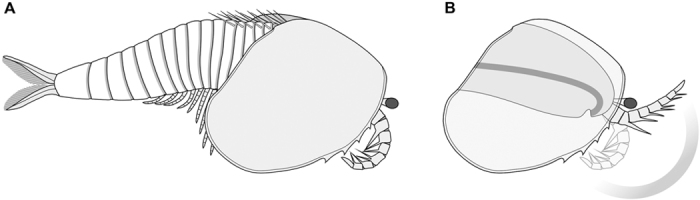
Morphological reconstruction of *Clypecaris serrata* sp. nov. (**A**) Complete body viewed from the right side. (**B**) Functional morphology of the raptorial appendage pair showing extended and flexed position for grasping food items. The proximity of the serrated anteroventral margins in the bivalved carapace relative to the flexed raptorial appendages suggests that both these structures were involved in feeding. The anteroventral flexure of the gut and position of the mouth opening are based on the cephalic organization of upper stem-group Euarthropoda^1^,^8^.

Complete individuals vary in sagittal length between 14–16 mm, and are consistently preserved flattened in oblique or dorsolateral view (Figs 1, 2, 3). The bivalved carapace covers ca. 50% of the trunk length (sag.), obscuring the morphology of the anterior body region (Figs 1A,H, 2A,C–E and 3B). The valves are approximately sub-oval in profile, and vary in size between 7–8 mm long (sag.) and 5–6 mm wide (transverse) between individuals (e.g. Figs 1A and 2E). The valves meet sagitally on the dorsal side of the trunk (Fig. 1A); the dorsal hinge represents approximately a third of the total carapace length (sag.), whereas the posterior rounded margin of the valves extend further posteriorly (Fig. 2E). The anteroventral margin of the valves is gently curved, and bears up to four short spines that confer a partially serrated appearance (Figs 1A,H, 2A,C–E and 3A). Each valve possesses a narrow elevated marginal rim (Figs 1A,H, 2C–E and 3A). The lateral eyes are bulbous and sit on flexible peduncles at the anterior end of the body (Fig. 2A–C); the bivalved carapace covers the proximal portion of both eyestalks and anterior edge of the head (Fig. 4). The first pair of appendages is situated in close proximity to the eyes. Each of the first appendages consists of eight podomeres with a cylindrical profile (Figs 1D–F,H–J, 2A,B and 3A). The most proximal podomere observable is the broadest (trans.) and lacks any projections (Fig. 1D,E). The second to seventh podomeres carry well-developed ventral spines that become progressively reduced in length towards the distal end of the limbs. Most podomeres carry a single spine that originates from the antero-ventral edge, in close proximity to the anterior margin of the corresponding article (Fig. 1D,E,I,J). The fifth podomere, however, differs in the possession of two spines instead of one. In all instances, the ventral spines are orientated at approximately 40–90^o^ relative to the main limb axis, facing towards the distal end of the limb in extended position (Fig. 4). The distal tip of the appendages – corresponding to the eighth podomere – is sub-conical and without spines. The articulated raptorial limbs evince considerable flexure in different specimens, indicating a broad range of motion (Figs 1A,D,E,H–J, 2A,B, 3AE and 4B). The proximal bases on the raptorial limbs are obscured by the bivalved carapace, and thus the precise organization at the anterior margin of the body is uncertain. The trunk has a sub-conical outline, and gently tapers towards the posterior end. The ring-like trunk tergites become more elongate (sag.) towards the posterior end (Figs 1A, 2A,C and 3B). Given that the carapace hinge is shorter than the length of the carapace, the trunk exposes a variable number of tergites ranging from some 13 (Fig. 2C) and up to more than 20 (Fig. 3B); approximately six or seven tergites are exposed dorsally on the anterior half of the trunk, each of these tergites bears a pair of delicate dorsal spines with a length of 1.5 mm (Figs 1A,B, 2C and 3B) that face postero-dorsally at an acute angle relative to the main body axis. Specimens preserved in oblique view demonstrate that each of the dorsal spines emerges from a rounded socket that is closely associated with the anterior edge of each of the trunk tergites (Fig. 3B,D). The anterior portion of the trunk is differentiated into a ‘thoracic’ region, in which each of the tergites bears a pair of delicate – possibly biramous – limbs that become progressively smaller towards the posterior end. The endopods have a slender construction; individual podomeres are not clearly visible (Figs 2C–E and 3B). Exopods cannot be clearly observed, with the exception of a single limb that is shorter and evinces a paddle-shaped outline (Fig. 3C). The last three trunk tergites are limbless, and form a discrete abdominal area (Figs 1A, 2A,D,E and 3B). The body terminates in a subconical telson that is longer (sag.) and narrower (trans.) than any of the preceding ring-like tergites (Figs 1A, 2A,C–E, 3B and 4A). An elongate pair of tail flukes (also referred to as ‘cerci’, ‘furcae’, ‘rami’, ‘uropods’, or ‘tail processes’ by different authors^15^,^16^,^17^,^18^,^19^,^20^,^21^,^22^,^23^,^24^) emerges at the posterior end of the trunk; each tail fluke narrows distally into an acute tip, giving it an acuminate outline, and is orientated at approximately 30^o^ relative to the main body axis (Figs 1G and 2C–F). The tail flukes articulate with the posterior base of the conical telson, are free throughout their length, do not overlap basally, and display a discrete longitudinal ridge that defines the outer margin (Fig. 2F). The inner margins of the tail flukes bear numerous elongate setae that face posteriorly (Figs 1G and 2F). There is some variation in the dimension of the tail flukes in different specimens, particularly regarding their proximal width (compare Figs 1G and 2F). Although these differences most likely reflect some intraspecific variation within the population, such as sexual dimorphism or ontogeny, it is not possible to further elaborate given the limited number of specimens with preserved tails. The digestive tract is the only part of the internal anatomy preserved in the available fossils (Figs 1A,H, 2C–E and 3B). The gut consists of a simple tube, approximately 400–700 μm wide (trans.), preserved with a distinctively three-dimensional profile. The presence of sediment in the gut is suggestive of early diagenetic permineralization and subsequent replacement by clay minerals, as posited for Chengjiang fossils^34^. Although the carapace obscures the anterior organization of the gut tract, the latter structure extends posteriorly into the telson, indicating that the anus is located terminally between the tail flukes (Fig. 1A,G,H).

### Comparisons with other Cambrian bivalved euarthropods

**Figure 5:**
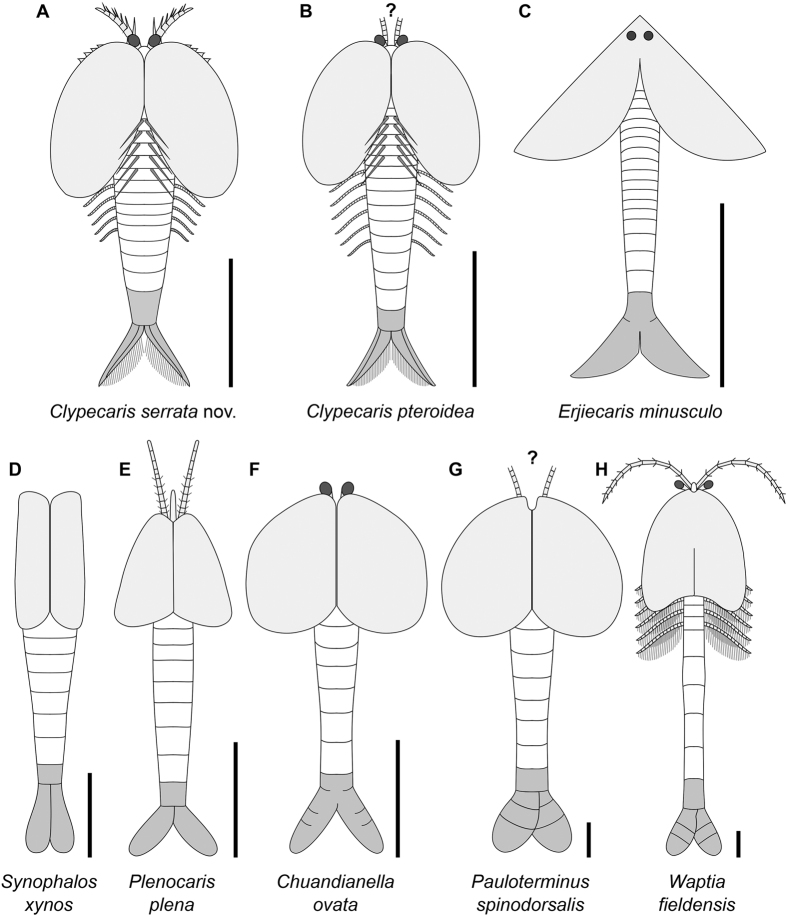
Morphological reconstructions of selected Cambrian bivalved stem-group euarthropods. Telson and tail flukes highlighted (*grey*) for comparison. Members of Clypecarididae (**A,B**) are distinguished by non-overlapping and acuminate tail flukes that bear straight setae on their inner margin. By contrast, Waptiidae (**D–H**) have dorsoventrally flattened tail flukes that overlap each other in various degrees, some with multiple articulations (**F–H**). (**A**) *Clypecaris serrata*, Stage 3 Xiaoshiba, China (this study). (**B**) *C. pteroidea*, Stage 3 Chengjiang, China^29^,^31^; reconstruction includes dorsal spines as implied by the presence of paired sockets on the trunk tergites. (**C**) *Erjiecaris minusculo*, Stage 3 Chengjiang, China^20^. (**D**) *Synophalos xynos*, Stage 3 Chengjiang, China^32^. (**E**) *Plenocaris plena*, Stage 5 Burgess Shale, Canada^40^. (**F**) *Chuandianella ovata*, Stage 3 Chengjiang, China^55^; (**G**) *Pauloterminus spinodorsalis*, Stage 3 Sirius Passet, Greenland^39^; (**H**) *Waptia fieldensis*, Stage 5 Burgess Shale, Canada^30^,^35^,^36^. Scale bars: 5 mm.

*Clypecaris serrata* evinces similarities with several bivalved stem-group euarthropods known from Cambrian deposits (Figs 4 and 5A). The best comparison can be made with the Chengjiang euarthropod *C. pteroidea*^29^,^31^ (Figs 3E and 5B). *C. serrata* closely resembles *C. pteroidea* in the overall shape and extent of trunk coverage of the bivalved carapace, the presence of paired stalked eyes, a tapering subconical trunk composed of ca. 20 ring-like tergites, slender trunk endopods, a limbless abdominal region consisting of three tergites plus a conical telson, paired non-overlapping acuminate tail flukes with longitudinal ridges and posteriorly facing setae, and simple tubular gut. New observations of *C. pteroidea* indicate the presence of paired dorsal sockets (Fig. 3E) – identical in their shape and distribution to those observed in *C. serrata* (Fig. 3B,D)– that imply the possession of similar spines to those observed the new taxon (Fig. 5B). The fundamental difference between both *Clypecaris* species is the possession of partially serrated anteroventral carapace margins in *C. serrata* (Figs 1A,C,H, 2A,C–E and 3A), whereas the valve margins of *C. pteroidea* are completely smooth (Fig. 3E). Given the incomplete preservation of the type material^29^,^31^, it is uncertain whether the first appendage pair of *C. pteroidea* had a raptorial construction as observed in *C. serrata* (Fig. 5A,B). Regardless of this complication, the close morphological parallels observed between *C. serrata* and *C. pteroidea* support their close phylogenetic relationship as members of Family Clypecarididae^29^. Both *Clypecaris* species somewhat resemble the bizarre euarthropod *Erjiecaris minusculo*^20^ in terms of general appearance and body tergite count, and particularly in the possession of acuminate tail flukes that do not overlap proximally (Fig. 5C). Other aspects of the body organization are strikingly different however, as *Erjiecaris* is distinguished by a uniquely partially fused bivalved carapace with a broad triangular outline, sessile dorsal eyes, and the absence of longitudinal ridges or setae on the tail flukes^20^. Thus, *Erjiecaris* may be a close relative of *Clypecaris*, but definitely not a member of Clypecarididae.

*Clypecaris* species also share a broad similarity with the waptiids, a loosely defined group of Cambrian bivalved euarthropods whose main defining feature is the possession of a paddle-like tail composed of a pair of partially overlapping dorsoventrally flattened flukes with rounded margins (Fig. 5D–H). *Clypecaris* species resemble waptiids in the presence of a bivalved carapace that covers the anterior body region, paired stalked eyes, delicate endopods, and a limb-less abdominal area (compare Fig. 5A,B with Fig. 5D–H). However, whereas waptiids have a paddle-like tail formed by dorsoventrally flattened flukes that overlap each other at their proximal bases (Fig. 5D–H), the flukes of *C. serrata* and *C. pteroidea* are elongate, do not overlap proximally, and bear numerous posterior-facing straight setae (Figs 1G, 2F and 5A,B). Some waptiids, such as *Waptia fieldensis*^30^,^35^,^36^,^37^, *Chuandianella ovata*^31^,^38^ and *Pauloterminus spinodorsalis*^39^ are also distinguished by the possession of multiarticulated tail flukes with three podomeres (Fig. 5F–H); intriguingly, the multiarticulated tail flukes are lacking in *Synophalos xynos*^32^ and *Plenocaris plena*^40^, which may inform on the distinction between these taxa and ‘legitimate’ waptiids. Waptiids are further distinguished from *Clypecaris* species by a trunk composed of fewer tergites, and the presence of an antenniform first appendage pair, as observed in *Plenocaris*^40^ (Fig. 5E), *Waptia*^35^,^36^ (Fig. 5H), and potentially also in *Pauloterminus*^39^ (Fig. 5G).

Comparisons with other Cambrian bivalved taxa are less phylogenetically informative, and mostly reflect symplesiomorphies of stem-group Euarthropoda. Both *Clypecaris* species broadly resemble *Canadaspis perfecta*^16^,^30^, *Perspicaris recondita*^15^ and *Perspicaris dictynna*^15^ in the possession of a carapace, stalked eyes and ring-like trunk tergites. However, these taxa differ from *Clypecaris* in having a more robust overall construction, antenniform first appendages with small paired setae on each podomere, a longer dorsal hinge, more elongate (sag.) valves, small marginal spines on the abdominal tergites borders, and tail flukes with setae on both the inner and outer margins^15^,^16^,^41^,^42^. Other relevant bivalved taxa include *Branchiocaris pretiosa*^41^,^43^, *Odaraia alata*^17^,^41^,^44^, *Pectocaris spatiosa*^29^, *Jugatacaris agilis*^18^, *Nereocaris exilis*^19^, *Nereocaris briggsi*^21^, and *Loricicaris spinocaudatus*^21^. These comparatively larger bivalved euarthropods are mainly distinguished from *Clypecaris* in the broader coverage of the carapace, and a body composed of dozens of trunk tergites, among other discrepancies in terms of head organization (e.g. anterior sclerite^44^) and posterior termination (e.g. morphology of tail flukes^21^). Among these bivalved stem-group euarthropods, the presence of a hook-like anteroventral process in the valves of *Nereocaris*^19^,^21^ evocates the serrated margin of *C. serrata* (Figs 1A,C,H, 2A,C–E and 3A); however, the lack of additional derived characters uniting these taxa suggests that the presence of valve hooks/serrations in these taxa is convergent.

The raptorial first appendage pair arguably represents one of the most distinctive characters of *C. serrata* (Figs 1D–F,H–J, 2A,B and 3A). Raptorial limbs are rare among bivalved stem-group euarthropods, as the first pair of limbs is generally expressed as a pair of uniramous sensorial antennae with several podomeres^8^,^21^,^35^,^36^ (Fig. 5). *Occacaris oviformis* and *Forfexicaris valida* both from the Chengjiang biota, are among the few Cambrian bivalved taxa with raptorial anterior appendages^29^,^31^. However, the limbs of *Occacaris* and *Forfexicaris* differ from *C. serrata* in that only two or four podomeres bear spines, and these are orientated towards the dorsal side instead of ventrally. These characteristics reflect a different functional morphology, even if the basic premise of grasping food items is similar (Fig. 4B). Although *Branchiocaris* has been described as a possessing a set of raptorial appendages^8^,^21^,^41^,^45^, these limbs lack spines, do not flex ventrally, and originate from a different segment altogether (i.e. second appendage pair). The only bivalved stem-group euarthropods that consistently display a raptorial first appendage are *Isoxys*^22^,^23^,^24^ and *Surusicaris*^46^. In these cases, however, the spine-bearing raptorial limbs are rotated such that they bend following an ascending motion. Thus, the broad functional similarities between the raptorial limbs of *C. serrata* relative to those of *Isoxys* and *Surusicaris* are best regarded as cases of convergent evolution. Raptorial limbs with multiple podomeres bearing ventral-facing spines are also observed in more distant total-group euarthropods, such as the radiodontans. The raptorial limbs of *C. serrata* resemble the radiodontan frontal appendages in terms of their overall construction and functional morphology. In particular, the presence of ventral facing spines with an anterior orientation in *C. serrata* is reminiscent of the spine construction in the radiodontans *Hurdia, Laggania* and *Amplectobelua*^47^. The frontal appendages of radiodontants differ greatly in terms of their podomere count and patterns of spine organization^46^,^47^, as well as their segmental origin from the protocerebral segment^1^, and thus their similarities with *C. serrata* are best regarded as a result of their common function.

## Discussion

### Phylogenetic affinities

**Figure 6:**
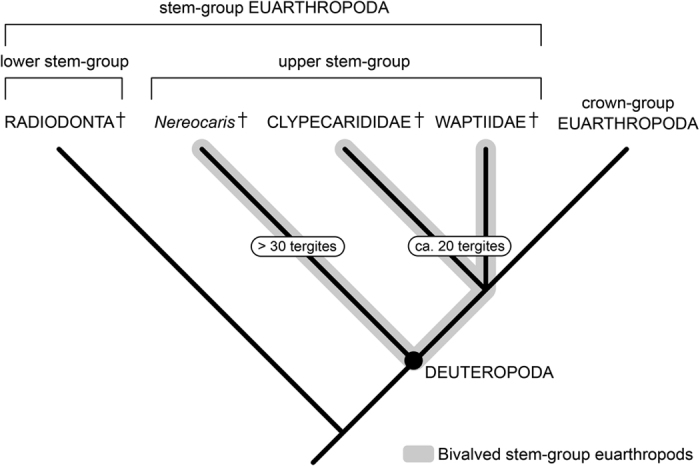
Simplified phylogeny of total-group Euarthropoda. Several major groups within the euarthropod stem-lineage (e.g. gilled lobopodians, fuxianhuiids, megacheirans) are not depicted for clarity (topology*, sans* Clypecarididae and Waptiidae, follows ref. 19). See ref. 1 for details of classification within total-group Euarthropoda. Clypecarididae, including *Clypecaris serrata*, and Waptiidae may occupy a crown-wards position relative to phylogenetically basal bivalved stem-euarthropods typified by long bodies with high tergites counts, such as *Nereocaris*^21^.

The presence of widespread body sclerotization, complete appendage arthropodization, and stalked lateral eyes followed posteriorly by the raptorial first appendage pair indicate that *Clypecaris serrata* can be reliably interpreted as a member of scion Deuteropoda (i.e. upper stem-group Euarthropoda + crown-group Euarthropoda; see ref. 1). Assigning the new taxon to a particular lineage, however, is more problematic. The appearance of *C. serrata* – and more broadly that of Cambrian bivalved forms (Fig. 5) – superficially resembles several carapace-bearing pancrustaceans, for example branchiopods and malacostracans. Indeed, early studies discussed Cambrian bivalved euarthropods in this general context^15^,^16^,^29^,^43^,^48^, and recent accounts have even argued that some of these taxa – such as waptiids – may be nested within the mandibulate crown-group^35^,^36^. The bivalved carapace of *C. serrata* obscures critical features of the anterior organization – particularly the structure of the post-oral appendages – and the lack of detailed post-cephalic appendicular data encumbers specific comparisons with members of crown-group Euarthropoda. In the absence of fine morphological detail, the similarities with bivalved Cambrian stem-group euarthropods represent the most reliable indicators for the affinities of *C. serrata*, and suggest that the new taxon is most likely a member of upper stem-group Euarthropoda (see topologies in refs. 1,12,21,45) (Fig. 6). Rather problematically, the phylogenetic position of *C. serrata* cannot be established more accurately, as *C. pteroidea* and waptiids have been consistently omitted from recent phylogenetic studies of Palaeozoic euarthropods^3^,^5^,^19^,^21^. Within upper stem-group Euarthropoda, bivalved forms with elongate bodies composed of numerous (commonly more than 30) short ring-like tergites (e.g. *Branchiocaris*^43^, *Odaraia*^17^*, Pectocaris*^29^, *Nereocaris*^21^, *Jugatacaris*^18^) have been consistently resolved as basal members of this lineage. If the presence of an elongate body with short tergites reflects a symplesiomorphic state among bivalved euarthropods as suggested by recent topologies^19^,^21^, the body organization of Clypecarididae would support a position closer to the euarthropod crown-group (Fig. 6). Following this logic, waptiids could potentially occupy an even more crownwards position relative to Clypecarididae given their apparently lower tergite count^32^,^35^,^36^,^39^,^40^; however, the precise relationships between the waptiids (Fig. 5D–H) and clypecaridids, including *C. serrata*, remain an open question pending a comprehensive revision of the phylogenetic of these extinct organisms.

### Functional morphology and palaeoecology

*Clypecaris serrata* features a distinctive combination of morphological adaptations that point towards an active predatory lifestyle (Fig. 4) the most striking of which is the structure of the first appendage pair. The presence of ventral-facing spines on most of the podomeres (Fig. 1A,D–F,I–J), coupled with the robust construction of these limbs relative to the trunk endopods (Figs 2D and 3B), indicate that they were adapted for a grasping function. The first appendages in *C. serrata* occupy the typical position of the deutocerebral limb pair in upper stem-group euarthropods^1^,^8^,^42^, and thus imply that these appendages had a pre-oral site of attachment to the body. The raptorial limbs would have operated by performing a downward stroke that brought the ventral-facing spines together, grasping the prey and bringing it to close proximity of the mouth (Fig. 4B). The construction of the raptorial limbs in *C. serrata* suggests that they worked in a similar way to the arthropodized frontal appendages of radiodontans (e.g. *Anomalocaris*, *Laggania, Hurdia*), as the latter also bear ventral facing spines that are used for grasping prey and bringing it close to the mouth opening^47^,^49^,^50^,^51^. The anteroventral position of the valve serrations in *C. serrata* also suggests their potential involvement in feeding. The acute end of the serrations faces posteriorly, and thus follows the orientation of the ventral spines in the raptorial appendages. We hypothesize that the serrations may have worked in tandem with the spine-bearing limbs to secure the prey close to the mouth during capture and feeding (Fig. 4B), which would imply that *C. serrata* sought after highly mobile prey that required to be forcibly immobilized prior to consumption. An alternative interpretation of the valve serrations of *C. serrata* as having a defensive function seems less likely, as the acute projections are rather short and would not effectively protect the body unless the animal was specifically attacked from the underside. Collectively, the available evidence suggests a specialized suite of adaptations for feeding, and tentatively offer novel insights into the otherwise limited understanding of Cambrian bivalved euarthropod palaeoecology. The interpretation of *C. serrata* as an active predator is consistent with its overall body construction, including mobile stalked eyes, delicate endopods and well-developed tail flukes with setae, which suggests that it had a primarily nektobenthic habitus. Finally, the presence of long paired dorsal spines on *C. serrata*, and strongly suggested in *C. pteroidea* based on the presence of paired dorsal sockets (Fig. 3E), is unique among Cambrian bivalved euarthropods (Fig. 5), and most likely served as a defensive mechanism against larger predators.

### Ecological implications

Investigations on the palaeobiology of the Xiaoshiba Konservat-Lagerstätten have yielded soft-bodied organisms with diverse palaeoecologies, including the sessile tube-dwelling worm *Selkirkia sinica*^52^, epibenthic mollusc-like animal *Wiwaxia foliosa*^25^, suspension feeding lobopodian *Collinsium ciliosum*^26^, and deposit feeding euarthropods (e.g. fuxianhuiids and trilobites)^8^,^27^. The recognition of an active nektobenthic predatory mode life for *Clypecaris serrata* reveals a previously unnoticed ecological niche for the Xiaoshiba biota, and thus contributes towards a more complete understanding of the multi-tiered community structure preserved in this important early Cambrian deposit. In a broader context, *C. serrata* reflects a greater diversity of feeding strategies explored by Cambrian bivalved euarthropods than previously considered. With the exception of *Isoxys*^23^,^24^ and *Surusicaris*^46^, most Cambrian bivalved forms have been tentatively regarded as either deposit or filter feeders^18^,^19^,^21^,^29^, or are too poorly known to gain significant insights about their autoecology. The predatory mode of life in *C. serrata* adds up to recent findings indicating that some Cambrian euarthropods explored niches that deviated from the palaeoecology of their close relatives, as also exemplified by the discovery of suspension/filter feeding-radiodontans^53^,^54^.

## Materials and Methods

Eleven specimens assigned to *Clypecaris serrata* sp. nov. were collected from the lowermost part of Hongjinshao Formation (Cambrian Stage 3) in eastern Kunming, China, where many exquisitely preserved fossils known as the Xiaoshiba Lagerstätten have been reported. According to the co-occurring trilobites, the fossil assemblage is within the uppermost part of the Qiongzhusian Stage^25^, existing approximately 515 million years ago. All specimens dealt with in this study are housed in the Key Laboratory for Palaeobiology, Yunnan University (YKLP).

Specimens were photographed by using a Leica M205C photomicroscope. All images were processed in Adobe Photoshop CS 4. Some of these digital photographs are accompanied by interpretative drawings to assist the interpretation of key features.

## Additional Information

**How to cite this article**: Yang, J. *et al.* A predatory bivalved euarthropod from the Cambrian (Stage 3) Xiaoshiba Lagerstätte, South China. *Sci. Rep.* **6**, 27709; doi: 10.1038/srep27709 (2016).
